# Supplementary figures and images for: Characterization of Nutritional Quality Traits of a Common Bean Germplasm Collection
Source: Foods. 2021 Jul 6;10(7):1572. doi: 10.3390/foods10071572 (PMC8306501; doi:10.3390/foods10071572)

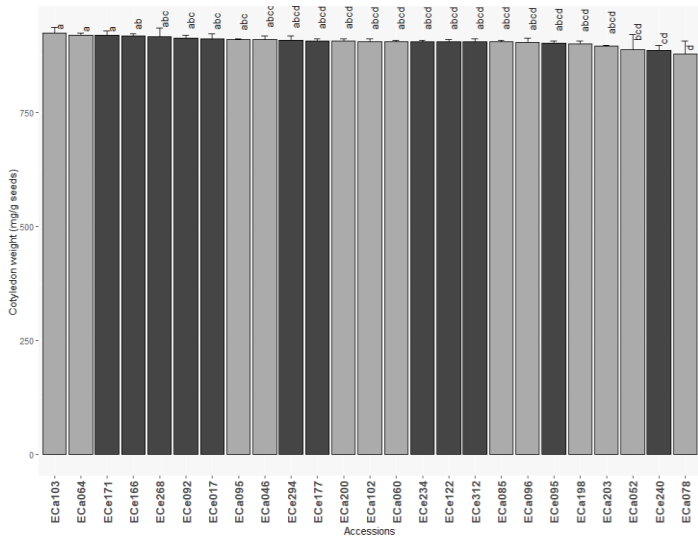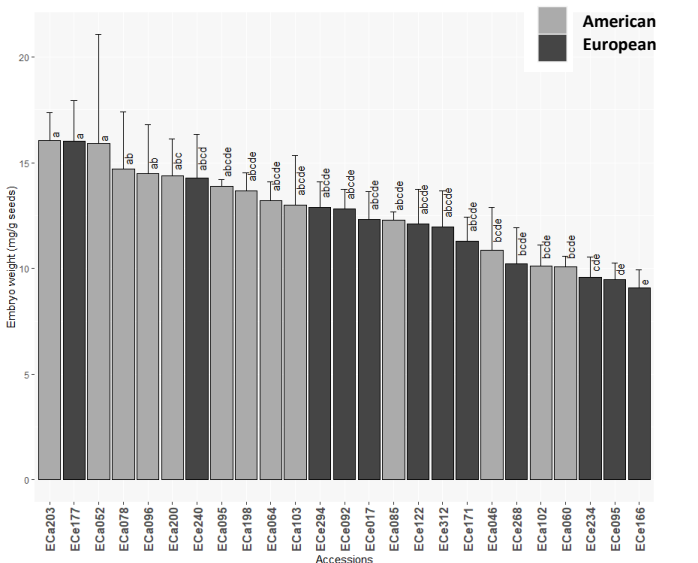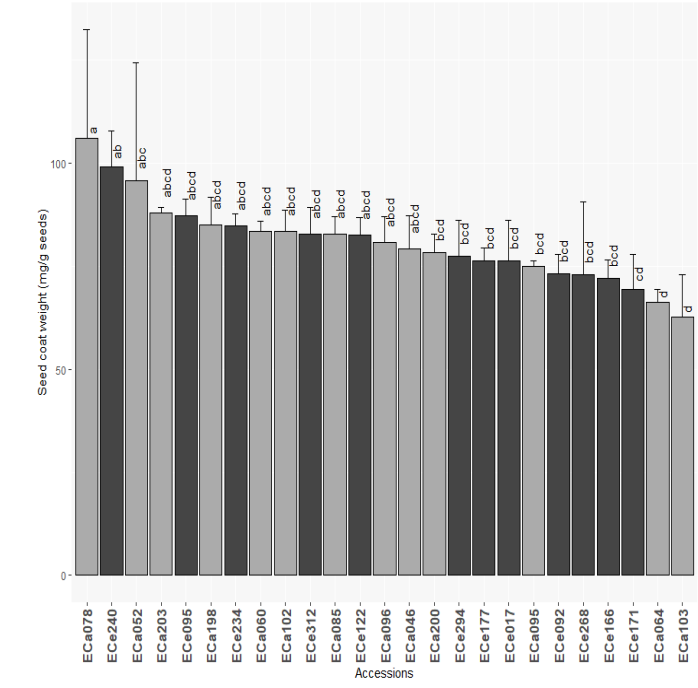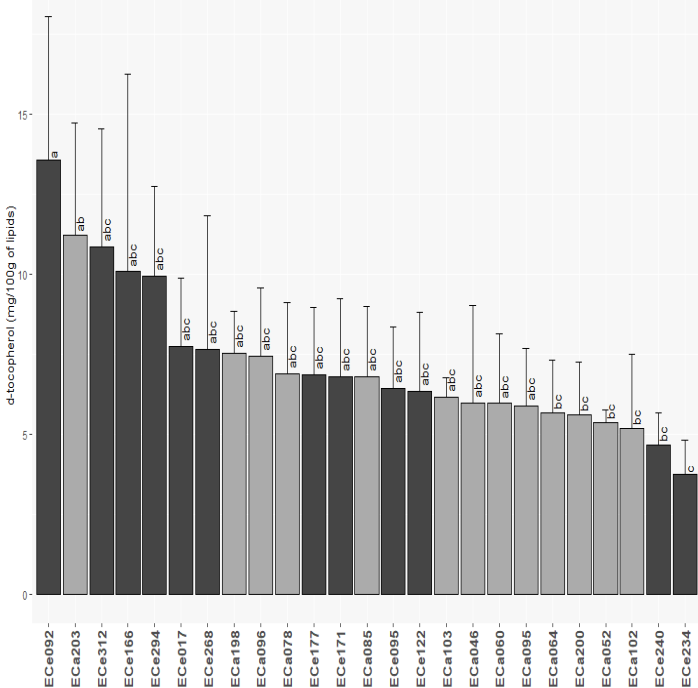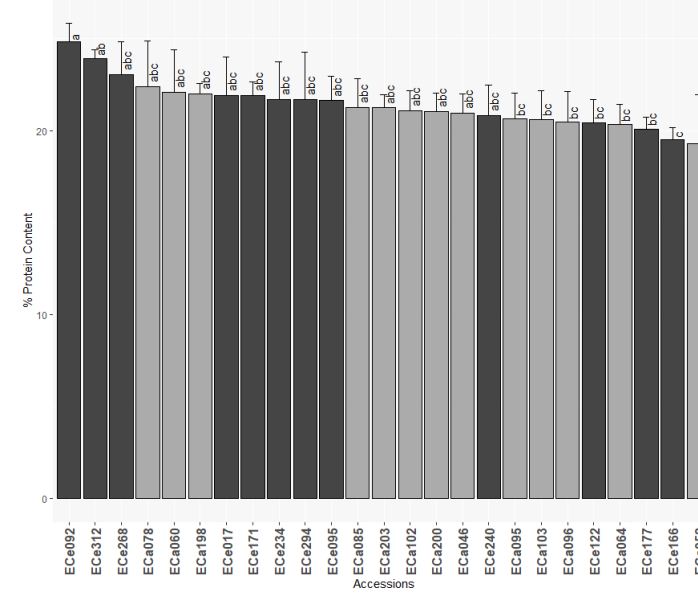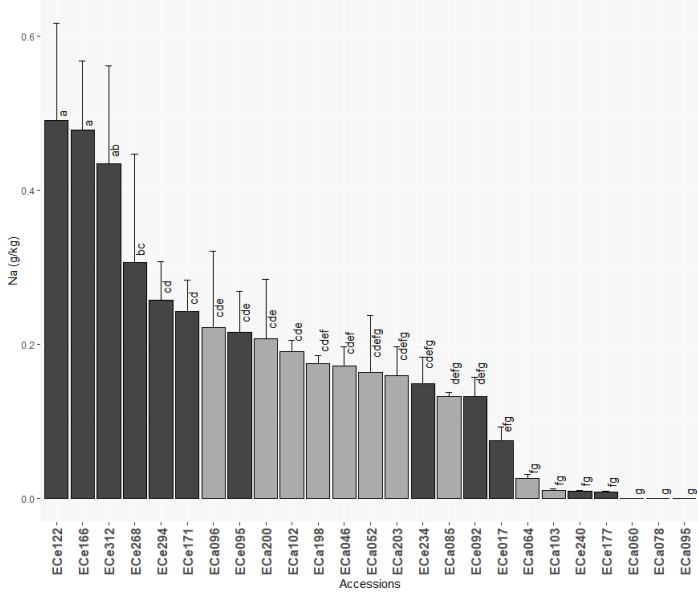

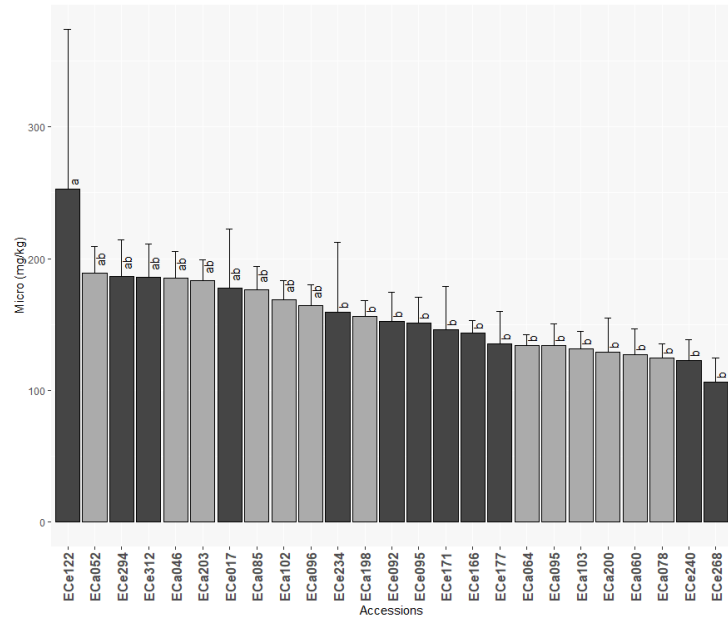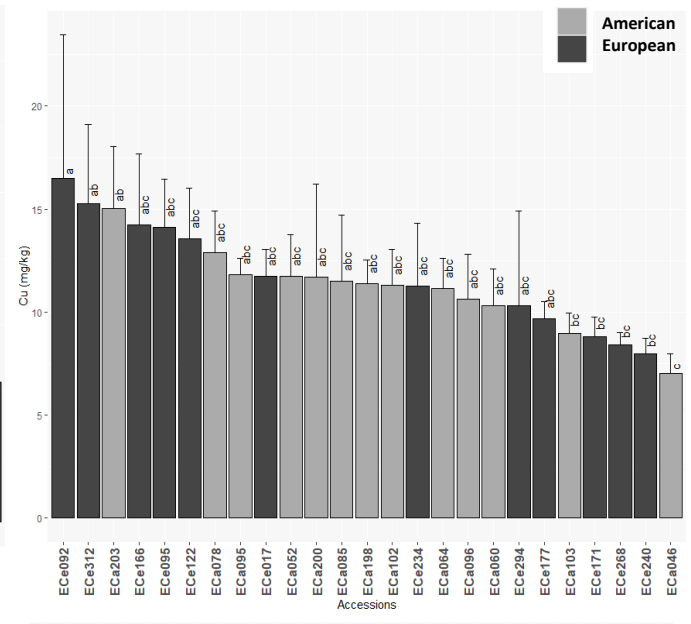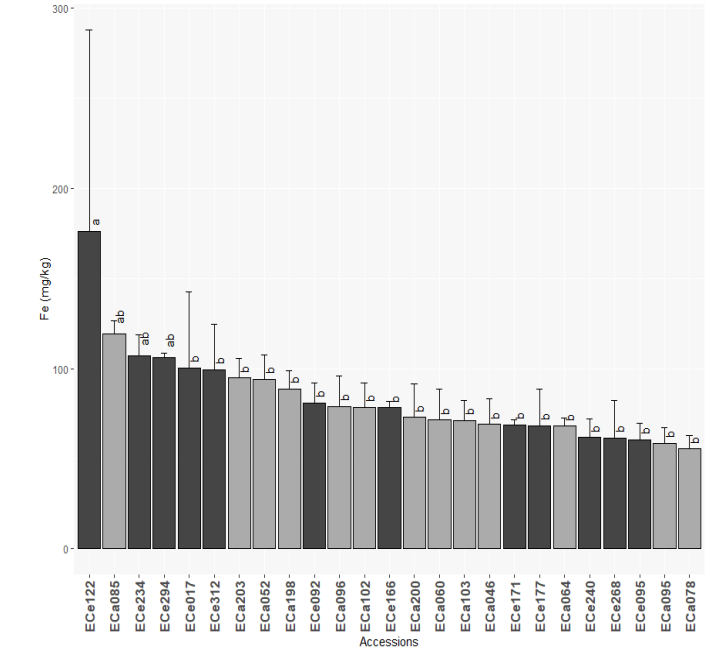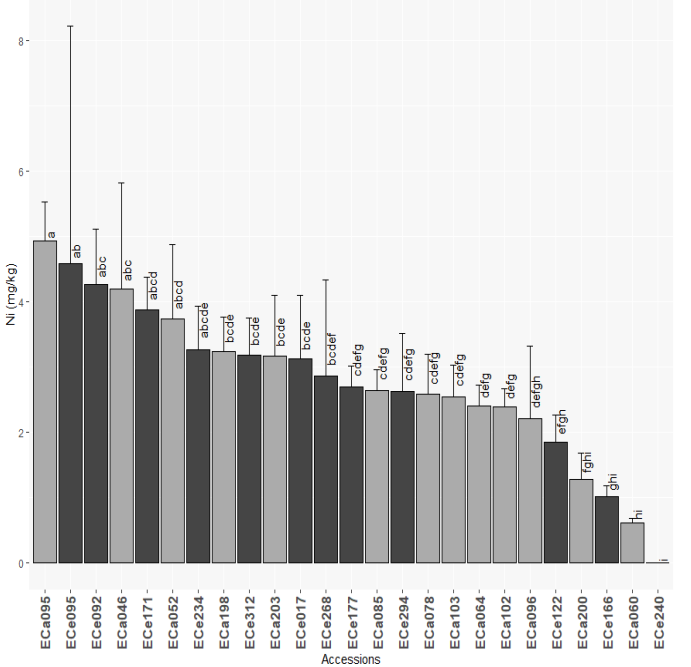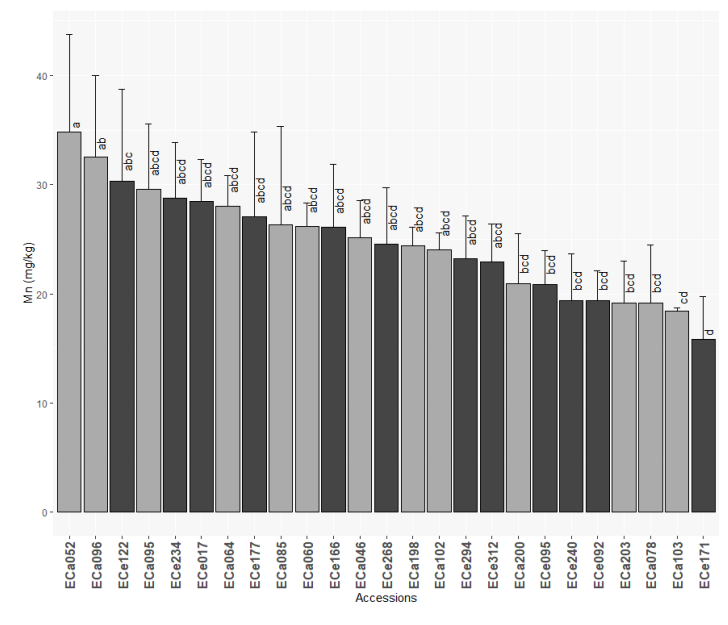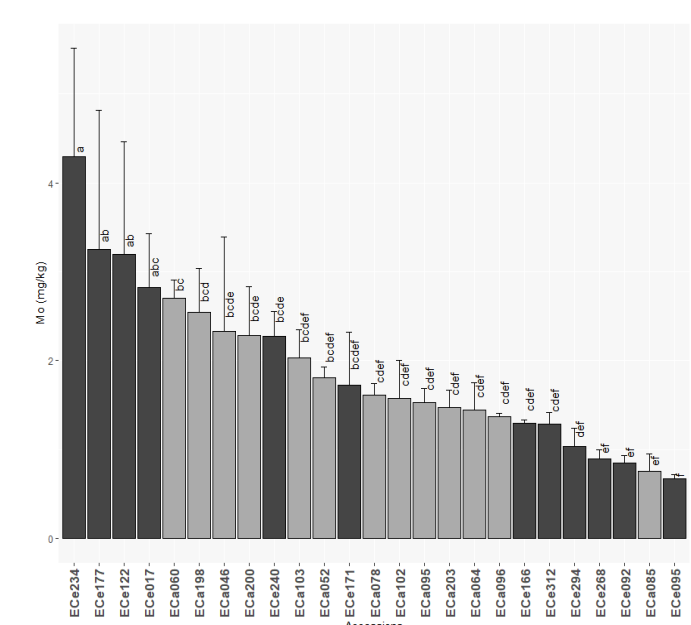

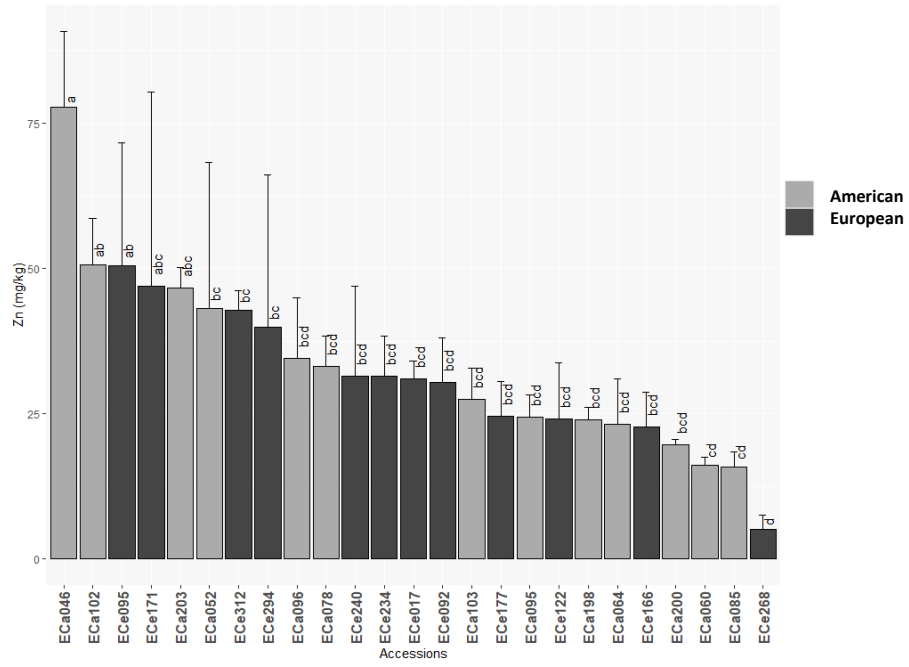

Supplement: Supplementary file 1 [file foods-10-01572-s001.zip › FigureS1.pdf]
